# CLN7 gene therapy: hope for an ultra-rare condition

**DOI:** 10.1172/JCI157820

**Published:** 2022-03-01

**Authors:** Jon J. Brudvig, Jill M. Weimer

**Affiliations:** Pediatrics and Rare Diseases Group, Sanford Research, Sioux Falls, South Dakota, USA.

## Abstract

CLN7 Batten disease, also known as variant late infantile neuronal ceroid lipofuscinosis type 7 (vLINCL7), is an ultra-rare form of Batten disease that presents early in life with severe neurological symptoms, including visual deficits, motor problems, and frequent seizures. There is high unmet need for disease-modifying therapies, as no existing treatment can halt progression or prevent premature death. In this issue of the *JCI*, Chen et al. present an AAV gene therapy for CLN7 that shows marked benefit in a mouse model of CLN7 Batten disease, paving the way for a phase I trial. The candidate gene therapy shows benefit for histopathology, behavioral abnormalities, and survival in mice and offers an acceptable safety profile in both mice and rats. Questions remain regarding dose, scaling, and timing of administration for patients, but this work is a substantial step forward for a very challenging disease.

## Batten disease

Batten disease is a family of neurodegenerative lysosomal storage disorders caused by mutations in 1 of at least 13 ceroid lipofuscinosis neuronal (*CLN*) genes (1). Pathogenesis generally initiates early in life, with cell-autonomous defects in intracellular trafficking and lysosomal function leading to neural dysfunction, neuroinflammation, neurodegeneration, and premature death. Unfortunately, etiology of most Batten disease subtypes is poorly understood, and this has hindered development of effective therapeutics (2). This lack of knowledge into relevant drug targets makes gene-replacement strategies particularly appealing. AAV-based gene therapies are currently under clinical development for Batten disease caused by mutations in the *CLN2* (3), *CLN3* (4–6), and *CLN6* (5, 7, 8) genes. In this issue of the *JCI*, Chen et al. expand this list to include *CLN7*, demonstrating considerable promise in preclinical studies (9).

CLN7 Batten disease, also known as variant late infantile neuronal ceroid lipofuscinosis type 7 (vLINCL7), is an ultra-rare form of Batten disease caused by biallelic mutations in *MFSD8* (*CLN7*), which encodes a putative lysosomal transporter of unknown function (10). Pathology is characterized by lysosomal storage material enriched in subunit C of mitochondrial ATP synthase, neuroinflammation, and neurodegeneration in various regions of the central nervous system, including the cerebral cortex, cerebellum, and retina (11). Affected individuals generally present early in life with severe neurological symptoms, including visual deficits, motor problems, and frequent seizures (10). Symptomatic treatments can offer some benefit, but there is no treatment that can halt progression or prevent premature death (2). Thus, there is high unmet need for disease-modifying therapies.

## AAV gene therapy for CLN7 Batten disease

**Figure 1:**
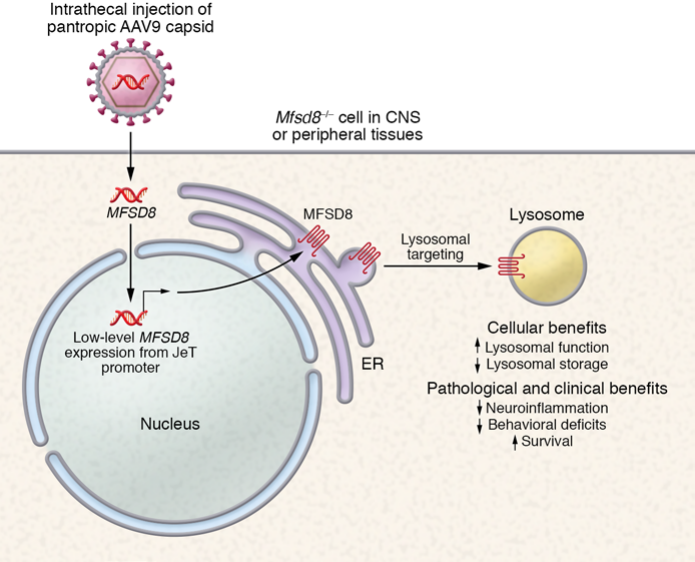
AAV9/*MFSD8* gene therapy for CLN7 Batten disease. In Chen et al.’s experimental gene therapy, intrathecal delivery of a pantropic AAV9 capsid resulted in expression throughout the CNS and peripheral tissues. Low-level expression of *MFSD8* was achieved with a JeT promoter element. Restoration of lysosomal *MFSD8* increased lysosomal enzyme activity and decreased lysosomal storage, resulting in a reduction in neuroinflammation, improvements in behavior, and extension of life span (9).

Chen et al. present an AAV gene therapy (Figure 1) that showed marked benefit in a mouse model of CLN7 Batten disease (9). The experimental therapy consists of an AAV9 capsid and a self-complementary AAV2 cassette expressing human *MFSD8* driven by a JeT promoter. This design is intended to ameliorate overexpression-mediated toxicity (e.g., dorsal root ganglia degeneration), as the relatively low level of expression driven by the synthetic JeT element should minimize the expression-related burden placed on transduced cells (12). Importantly, the authors demonstrate that this low-level expression sufficiently rescued lysosomal phenotypes in patient fibroblasts and that a stronger promoter offered no improvement in efficacy.

The authors present an impressively comprehensive efficacy study in *Mfsd8^–/–^* mice, testing high (5 × 10^11^ vector genome [vg]/mouse) and low (1.25 × 10^11^ vg/mouse) doses administered intrathecally at pre- and postsymptomatic time points. AAV9/*MFSD8* improved survival in a manner that was dependent on both dose and timing of administration, with the best benefit obtained from a high dose delivered early in life (median survival 16.8 months vs. 7.8 months for untreated). Pathological analyses thus focused on the presymptomatically dosed cohort, finding again that the higher dose offered more widespread *MFSD8* expression and superior efficacy for substrate reduction and amelioration of neuroinflammation. Behavioral phenotypes are generally mild in *Mfsd8^–/–^* mice, but here too, early treatment with a high dose optimally stabilized motor function.

This efficacy data were complemented by safety data from a non–good laboratory practice (non-GLP) study in wild-type mice and a GLP study in wild-type rats, both of which demonstrated an acceptable safety profile. Collectively, these studies closely followed the established blueprint for an investigational new drug (IND) application with the FDA. Indeed, the authors referenced IND approval for their AAV9/*MFSD8* vector as of December 2020 and subsequent initiation of a phase 1 trial to evaluate clinical safety and efficacy. For this trial, the authors proposed a top dose of 1 × 10^15^ total vg, a relatively high dose that nevertheless corresponds to roughly half of the high dose in the mouse efficacy study when scaled based on mature cerebrospinal fluid (CSF) volumes.

## Conclusions and clinical considerations

As with all first-in-human gene-therapy studies, suitability of this dose extrapolation will be a key question going into the clinical trial. On a linear basis, the proposed dose is marginally lower than the most efficacious dose tested in mice, but species differences unfortunately suggest that efficiency of gene replacement declines with increasing body size, favoring even higher doses than those suggested by linear models (13, 14). Chen et al. (9) also lacks any characterization of biodistribution in nonhuman primates, which are believed to offer the best approximation of gene transfer efficiency in humans, further impeding dose extrapolation. Thus, while the proposed 1 × 10^15^ total vg dose offers a conservative safety margin and a reasonable hope for clinical efficacy, it is likely that an optimally efficacious dose would be considerably higher. Unfortunately, as the authors suggest, manufacturing considerations most commonly dictate what is achievable for a top dose in clinical studies, as intrathecal administration is limited by volume and total doses are thus dependent on vector prep concentration. AAV9 vectors tend to aggregate when concentrated beyond about 1 × 10^14^ capsid particles/mL, and this practical limitation has surely been a determinant of top doses in trials for many CSF-administered gene therapies (15, 16).

The Chen et al. (9) results also highlight a key dependency for efficacy on age of dosing. In *Mfsd8^–/–^* mice, dosing early in life at postnatal days 7 to 10 conferred nearly three times the life span extension as compared with dosing at postnatal day 120. Dosing at 6 months of age offered no life span extension. Unfortunately, it is unclear exactly what these results may suggest for individuals living with CLN7 Batten disease. Approximations of age equivalency between mice and humans are rough at best and are complicated even further by poorly understood differences in the timing of disease progression. In any case, the inverse relationship between age of dosing and subsequent efficacy supports what has long been assumed for pediatric neurodegenerative disorders — prevention of pathology and symptom development is more feasible than stabilization, which is more feasible than reversal. In a disease such as CLN7 Batten, wherein cell-autonomous defects lead to an aggressive cascade of neuronal dysfunction, neuroinflammation, and neurodegeneration (11), disease-modifying therapies such as this one will be most efficacious when deployed as early as possible in disease progression.

The limitations of the present study in no way undermine what is a notably thorough body of work. Rather, they highlight key challenges encountered across the gene-therapy space: Even the best animal models are remarkably lacking. We have an immature understanding of dose scaling. And we are rarely able to treat patients as early as we would like. Still, work such as Chen et al. (9) paves the way for safe clinical trials with a reasonable expectation of efficacy and initiates what may be a long road ahead for clinical optimization of the therapy. For families affected by CLN7 Batten disease, who face the certainty of severe disease and unrelenting progression, this study also provides much needed hope.

## Version 1 03/01/2022

Electronic publication

## References

[1] Johnson TB (2019). Therapeutic landscape for Batten disease: current treatments and future prospects. Nat Rev Neurol.

[2] Brudvig JJ, Weimer JM (2021). On the cusp of cures: breakthroughs in Batten disease research. Curr Opin Neurobiol.

[3] Sondhi D (2020). Slowing late infantile Batten disease by direct brain parenchymal administration of a rh.10 adeno-associated virus expressing *CLN2*. Sci Transl Med.

[4] Bosch ME (2016). Self-complementary AAV9 gene delivery partially corrects pathology associated with juvenile neuronal ceroid lipofuscinosis (CLN3). J Neurosci.

[7] Cain JT (2019). Gene therapy corrects brain and behavioral pathologies in CLN6-Batten Disease. Mol Ther.

[9] Chen X (2022). AAV9/MFSD8 gene therapy is effective in preclinical models of neuronal ceroid lipofuscinosis type 7 disease. J Clin Invest.

[10] Kousi M (2009). Mutations in CLN7/MFSD8 are a common cause of variant late-infantile neuronal ceroid lipofuscinosis. Brain.

[11] Damme M (2014). Gene disruption of Mfsd8 in mice provides the first animal model for CLN7 disease. Neurobiol Dis.

[12] Tornøe J (2002). Generation of a synthetic mammalian promoter library by modification of sequences spacing transcription factor binding sites. Gene.

[13] Aksenov S (2021). Current and next steps toward prediction of human dose for gene therapy using translational dose-response studies. Clin Pharmacol Ther.

[14] Tang F (2021). Rational clinical dose selection of adeno-associated virus-mediated gene therapy based on allometric principles. Clin Pharmacol Ther.

[15] Qu G (2003). 901. Evidence that ionic interactions are involved in concentration-induced aggregation of recombinant adeno-associated virus. Molecular Therapy.

[16] Wright JF (2013). Concentration of AAV8 and 9 vector to titers ranging from 1-6x1^01^4 vg/ml: Feasibility assessment for volume-limited dosing. Molecular Therapy.

